# Comparison of a Two (32/38 Weeks) versus One (36 Weeks) Ultrasound Protocol for the Detection of Decreased Fetal Growth and Adverse Perinatal Outcome

**DOI:** 10.3390/jpm14070709

**Published:** 2024-07-01

**Authors:** Mar Nieto-Tous, Blanca Novillo-Del Álamo, Alicia Martínez-Varea, Elena Satorres-Pérez, José Morales-Roselló

**Affiliations:** 1 Departamento de Obstetricia y Ginecología, Hospital Universitari i Politècnic La Fe, 46026 Valencia, Spain; novillo_bla@gva.es (B.N.-D.Á.); martinez_alivar@gva.es (A.M.-V.); satorres_ele@gva.es (E.S.-P.); jose.morales@uv.es (J.M.-R.); 2 Departmen of Medicine, CEU Cardenal Herrera University, 12006 Castellón de la Plana, Spain; 3 Faculty of Health Sciences, Universidad Internacional de Valencia, 46002 Valencia, Spain; 4 Instituto de Investigación Sanitaria La Fe de Valencia, Hospital Universitari i Politècnic La Fe, 46026 Valencia, Spain; 5 Departamento de Pediatría, Obstetricia y Ginecología, Facultad de Medicina, Universitat de València, 46010 Valencia, Spain

**Keywords:** small for gestational age, adverse perinatal outcome, low-risk pregnancy, third-trimester, ultrasound

## Abstract

Third-trimester ultrasound has low sensitivity to small for gestational age (SGA) and adverse perinatal outcomes (APOs). The objective of this study was to compare, in terms of cost-effectiveness, two routine third-trimester surveillance protocols for the detection of SGA and evaluate the added value of a Doppler study for the prediction of APO. This was a retrospective observational study of low-risk pregnancies that were followed by a two growth scans protocol (P2) at 32 and 38 weeks or by a single growth scan at 36 weeks (P1). Ultrasound scans included an estimated fetal weight (EFW) in all cases and a Doppler evaluation in most cases. A total of 1011 pregnancies were collected, 528 with the P2 protocol and 483 with the P1 protocol. While the two models presented no differences for the detection of SGA in terms of sensitivity (47.89% vs. 50% *p* = 0.85) or specificity (94.97 vs. 95.86% *p* = 0.63), routine performance of two growth scans (P2) led to a 35% cost increase. The accuracy of EFW for the detection of SGA showed a noteworthy improvement when reducing the interval to labor, and the only parameter with predictive capacity of APO was the cerebroplacental ratio at 38 weeks. In low-risk pregnancies, the higher costs of a two-scan growth surveillance protocol at the third trimester are not justified by an increase in diagnostic effectivity.

## 1. Introduction

One of the major goals in obstetrics is the reduction in adverse perinatal outcomes (APOs) through the detection of fetal compromise prior to delivery. APO is known to have more of an impact in small-for-gestational-age (SGA) fetuses (defined as birthweight (BW) < 10th centile) [1], as they are more affected by uteroplacental insufficiency. Therefore, the ultrasonographic diagnosis of SGA (estimated fetal weight (EFW) < 10th centile) is commonly used as an approximation of fetal growth restriction (EFW < 10th centile plus hypoxic derived Doppler changes or EFW < 3rd centile), a concept that is more difficult to gauge because it defines the failure of the fetus to achieve its growth potential [2].

Third-trimester ultrasonography and EFW present low sensitivity for SGA [3,4,5,6], leading to an ongoing debate regarding its ability to reduce APO. However, universal screening in low-risk pregnancies might improve its performance [7]. This becomes relevant considering that the failure to identify these fetuses antenatally might be related to a 1.6 to 4.3 fold increase of fetal and perinatal death [1,8]. Regarding the timing of universal third-trimester ultrasonography, previous studies [9,10] suggest that, in general terms, the sensitivity increases with a lower interval to delivery [11].

Moreover, although EFW might be useful to screen for fetuses at a higher risk of abnormal growth and APO [7], it fails to identify suboptimal growth in appropriate-for-gestational-age (AGA) fetuses growing over the 10th centile, which could account for as much as two thirds of neonatal complications secondary to hypoxia at term [12,13]. This has encouraged the identification of additional Doppler markers for placental insufficiency, like the cerebroplacental ratio (CPR) [14].

Therefore, the aim of this study was to compare, in terms of cost-effectiveness, two routine third-trimester surveillance protocols for the detection of fetal smallness, evaluating their accuracy according to the interval to labor. In addition, we also assessed the added value of Doppler for the detection of fetuses at risk of APO.

## 2. Materials and Methods

### 2.1. Study Design

We performed a retrospective observational study of pregnancies at the La Fe hospital fetal ultrasound unit for routine third-trimester follow-up between 2012 and 2022. This interval presented two periods with different surveillance scan protocols. The surveillance protocol between 2012 and 2018 included two growth scans at 32 and 38 weeks (P2), while in that between 2019 and 2022, this protocol was reduced to a single growth scan at 36 weeks (P1). In both protocols, a further ultrasound scan was performed at 40 weeks, close to the estimated delivery date. During both periods, the hospital delivered about 5.000 babies per year, and the sample to study was calculated in 850, considering that during the study period, 10% of the approximately 50.000 fetuses delivered were SGA, with a confidence level of 95% and a statistical power of 80%.

Gestational age (GA) was initially determined according to the crown–rump length at the first-trimester ultrasound. The inclusion criteria were women between 18 and 40 years, with singleton pregnancies, no risk factors, and the absence of fetal anomalies at the first- and second-trimester ultrasound scans. Exclusion criteria were a history of diabetes (pre-gestational or gestational), hypertensive disorders, stillbirth, thrombophilia, autoimmune or renal disease, and any other condition leading to a higher risk of growth disorders.

Ultrasound scans included an estimated fetal weight (EFW), and in most cases a Doppler evaluation of the umbilical (UA) and middle cerebral arteries (MCA) pulsatility indices (PI), which were used to calculate the cerebroplacental ratio (CPR) (MCA PI/UA PI). Doppler measures were taken during maternal quiescence, in the absence of fetal breathing movements and tachycardia, and keeping the insonation angle with the examined vessel as small as possible. The UA PI was obtained in a free loop of the umbilical cord, while the MCA PI was obtained at the MCA portion adjacent to the sphenoid wing.

To adjust the effect of gestational age, the EFW and BW values were converted into centiles using local population charts adjusted for fetal gender [15], while Doppler values were converted into multiples of median (MoM), dividing each value by the 50th centile at each gestational age [16,17]. The medians of UA PI, MCA PI, and CPR are represented by the following equations:

MCA 50th centile = –3.266164164 + 0.368135209 ∗ GA (in weeks) − 0.005251488 ∗ GA (in weeks)

UA 50th centile = 2.2037 − 0.057955 ∗ GA (in weeks) + 0.00053953 ∗ GA (in weeks)

While the CPR was represented by this other equation:

CPR 50th centile = –3.814786276 + 0.36363249 ∗ GA (in weeks) − 0.005646672 ∗ GA (in weeks).

The ultrasound scans were performed by experts in obstetric ultrasound, using General Electric Voluson^®^ (E8/E6/730) machines equipped with 2–8 MHz convex proofs.

Neonatal data, including GA at delivery, interval examination labor, BW, onset of labor, via of delivery, 5 min Apgar scores, cord arterial pH, and admission to the neonatal ward or intensive care unit, were collected after birth.

SGA was considered when BW was below the 10th centile, while adverse perinatal outcome (APO) was defined using a composite outcome with four components: (1) Cesarean section for abnormal intrapartum cardiotocogram (CTG) (according to the intrapartum fetal monitoring guidelines of the FIGO) [18] or intrapartum fetal scalp pH < 7.20; (2) neonatal arterial umbilical cord pH < 7.10; (3) five-minute Apgar score < 7; and (4) postpartum admission to neonatal ward or intensive care unit. Subsequent derived scans were defined as those that were programmed after the first scheduled scan was performed. The onsets of labor occurred for obstetric indications and fetuses were managed, according to their progression at labor, following the hospital protocol.

### 2.2. Statistical Analysis

Continuous descriptive statistics, represented by the mean plus SD, median and interquartile range (1st and 3rd quartile), were calculated for maternal age, gravidity, parity, maternal pre-pregnancy weight, maternal height, maternal body mass index, GA at the examination in weeks, GA at delivery in weeks, and BW and BW centile, while categorical descriptive statistics represented by the number (N) and frequency (%) were calculated for nulliparity, smoking, fetal gender, Caucasian ethnicity, type of labor onset (spontaneous, induction or elective cesarean section), method of delivery (spontaneous or assisted vaginal delivery, elective or for failure to progress or for abnormal CTG cesarean section), 5 min Apgar score < 7, arterial pH < 7.10, neonate destiny (maternal or neonates ward or pediatric intensive care unit), adverse perinatal outcome, abnormal CTG, and small for gestational age (SGA) (<10th centile).

The accuracy of the EFW centile for the detection of SGA and APO was evaluated separately for each of the study weeks (32, 36, 38, and 40) using ROC curves with their area under the curve (AUC) and detection rate (DR) for a false positive rate (FPR) of 5% and 10%. In addition, accuracy was also calculated for each of the entire scan protocols: one scan (36 weeks) versus two scans (32/38 weeks) using the sensitivity, specificity, positive likelihood ratio, and negative likelihood ratio of each procedure. Finally, in cases where Doppler data (UA PI, and CPR) were available, their accuracy for the detection of SGA and APO was again calculated for each of the study weeks (32, 36, 38 and 40 weeks) by means of ROC curves with their AUC, and DR for a FPR of 5% and 10%.

Comparisons between groups were made with Mann–Whitney U tests for continuous data and Fisher’s exact tests for categorical data. Statistical analysis and charts were performed with Stat Plus^®^ 7 and GraphPad Prism^®^ 9 for Apple Macintosh. Significance was established at a *p* < 0.05. IRB permission was obtained for the study (Reference 2022-157-1). The authors report no conflicts of interest.

## 3. Results

### 3.1. Study Population

A total of 1011 pregnancies were collected, 528 with the P2 protocol (at 32 and 38 weeks) and 483 with the P1 protocol (at 36 weeks). The characteristics of the study population are shown in Table 1. The mean maternal age was 31.61 years, and the mean pre-pregnancy body mass index was 23.94. Almost half of the women were nulliparous (49.26%), most of them Caucasian (95.35%), and only 12.66% smoked during pregnancy. Concerning delivery, most pregnancies presented a spontaneous onset of labor (57.27%) and a spontaneous (60.83%) or assisted vaginal delivery (19.58%), and most neonates were admitted to the maternal ward (94.06%). In addition, only 0.3% presented an Apgar score below 7 at 5 min, only 2.67% had an arterial pH below 7.10, and only 4.95% were admitted at the neonatal ward or pediatric intensive care unit. Finally, the proportion of SGA fetuses (<10th percentile), was 11.77% and the frequency of APO was 11.17%.

The characteristics of the study population according to the specific ultrasonographic surveillance protocol are shown in Table 2. In summary, women following P2 were more frequently of Caucasian origin (97.35% versus 3.17%, *p* < 0.0024), while women in P1 had a higher proportion of labor induction (41.41%, versus 32.77%, *p* < 0.005) and a higher frequency of arterial pH below 7.10 (3.93% versus 1.52%, *p* < 0.019). No differences between groups were found in the frequency of SGA and APO.

### 3.2. Comparison of P1 and P2 in Terms of Cost-Effectiveness

The overall efficacy of P2 and P1 models presented no differences in the detection of SGA in terms of sensitivity (47.89 vs. 50% *p* = 0.85), specificity (94.97 vs. 95.86% *p* = 0.63), positive likelihood ratio (59.57 vs. 57,14% *p* = 0.84), or negative likelihood ratio (92.14 vs. 94.56% *p* = 0.18). However, the routine performance of a two-scan instead of a one-scan surveillance protocol represented a notable increase in the number of ultrasounds and consequently in the economic burden, with no proven increase in the detection rate of SGA. Assuming an estimated cost of 100 euros per ultrasound and considering that the scheduled and subsequent derived scans led to an average of 1.68 (range 1–4) scans in the P1 and 2.57 (range 1–6) scans in P2, the average total cost per pregnancy would be EUR 167.9 in the case of P1 and a 35% increase, EUR 256.6, in the case of P2.

### 3.3. Accuracy of EFW and Doppler According to the Interval to Labor

**Figure 1:**
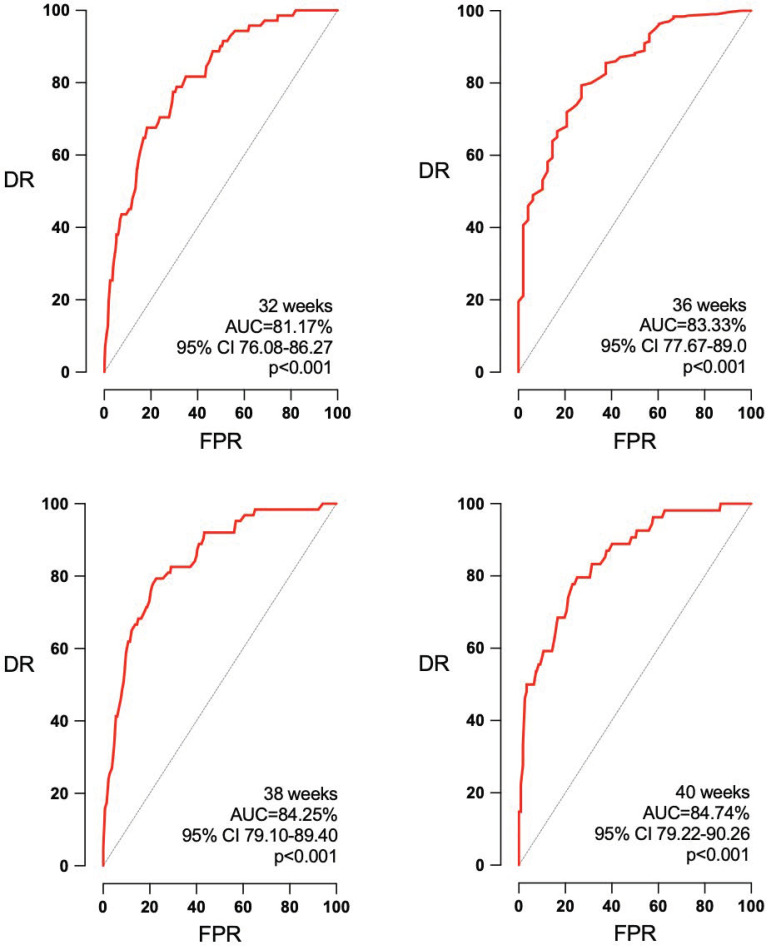
Improvement in the accuracy of EFW for the detection of SGA with the reduction in the interval to labor. AUC: area under the curve, CI: confidence interval, DR: detection rate, FPR: false positive rate, *p*: *p* value.

**Figure 2:**
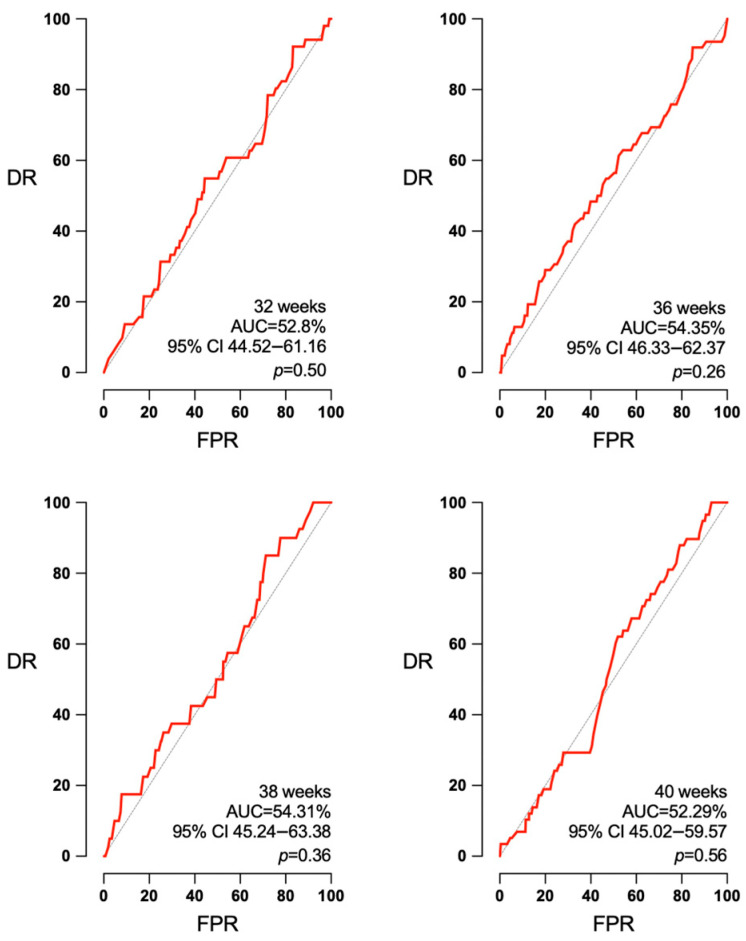
Absence of predictive capacity of EFW for APO in any interval to delivery. AUC: area under the curve, CI: confidence interval, DR: detection rate, FPR: false positive rate, *p*: *p* value.

Concerning the accuracy of EFW for the detection of SGA and APO according to the interval to labor, a small but noteworthy improvement was observed for the AUC of SGA at 32, 36, 38, and 40 weeks (Figure 1): 81.17% (95% confidence interval (CI) 76.08–86.27%, *p* < 0.001), 83.33% (95% CI 77.67–89.0%, *p* < 0.001), 84.25% (95% CI 79.10–89.40%, *p* < 0.001), and 84.74% (95% CI 79.22–90.26%, *p* < 0.001). However, EFW was not predictive of APO at any of the intervals to delivery (Figure 2): AUC 52.8 (95% CI 44.52–61.16, *p* = 0.50), 54.35 (95% CI 46.33–62.37, *p* = 0.26), 54.31 (95% CI 45.24–63.38, *p* = 0.36), and 52.29 (95% CI 45.02–59.57, *p* = 0.56) for 32, 36, 38, and 40 weeks.

**Figure 3:**
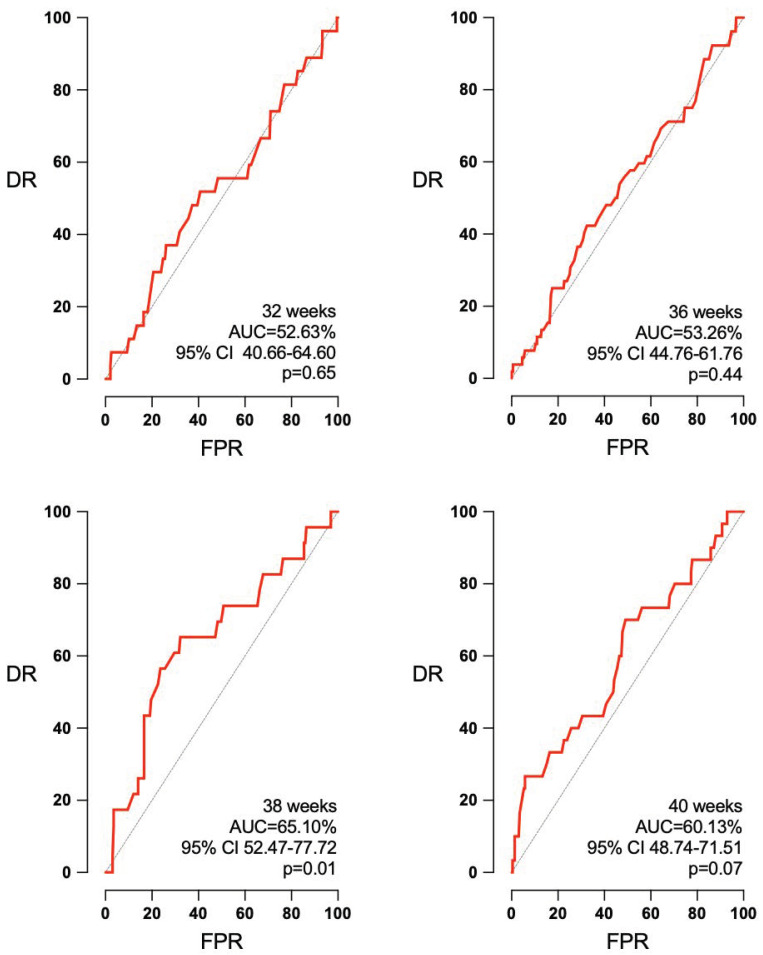
CPR’s predictive capacity of APO according to the interval to delivery. AUC: area under the curve, CI: confidence interval, DR: detection rate, FPR: false positive rate, *p*: *p* value.

**Figure 4:**
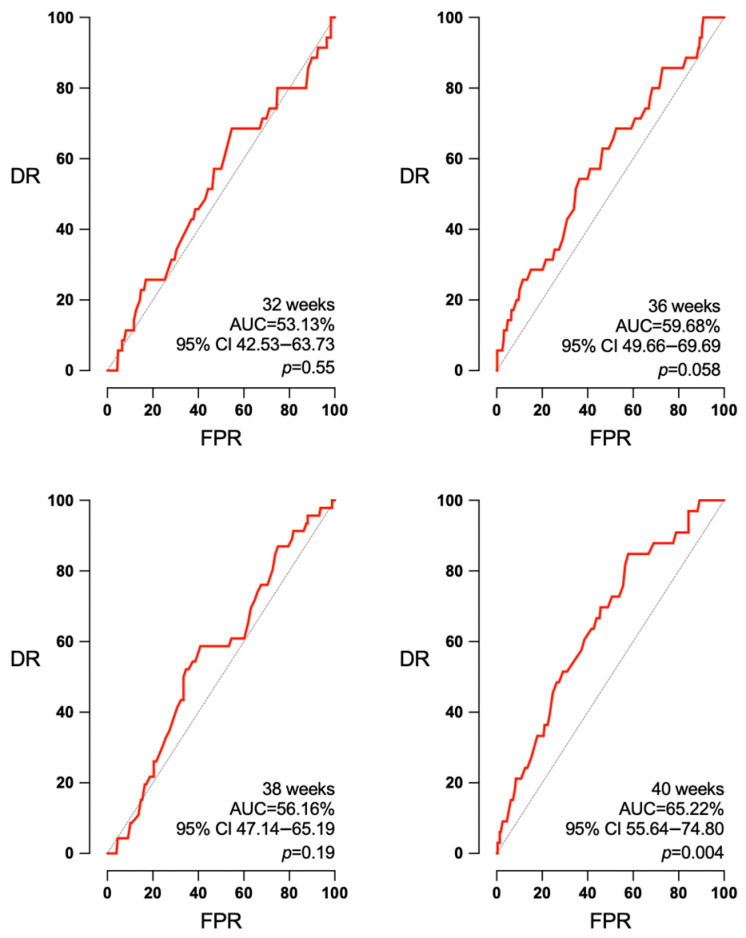
CPR’s predictive capacity of SGA according to the interval to delivery. AUC: area under the curve, CI: confidence interval, DR: detection rate, FPR: false positive rate, *p*: *p* value.

Concerning Doppler ultrasound, CPR and UA PI measurements were obtained in an important number of pregnancies: 256 and 302 at 32 weeks, 400 and 471 at 36 weeks, 222 and 273 at 38 weeks, and 256 and 454 at 40 weeks. Figure 3 shows the ability of CPR to predict APO at the four studied gestational ages. At 38 weeks, CPR was able to predict APO (AUC 65.10, 95% CI 52.47–77.72%, *p* = 0.01). However, no predictive ability was observed at 32 weeks (AUC 52.63, 95% CI 40.66–64.60%, *p* = 0.65), 36 weeks (AUC 53.26, 95% CI 44.76–61.76%, *p* = 0.44), or 40 weeks (AUC 60.13, 95% CI of 48.74–71.51%, *p* = 0.07). CPR was also predictive of SGA at 40 weeks, AUC 65.22 (95% CI 55.64–74.80%, *p* = 0.004), but not at 32 weeks, AUC 53.13 (95% CI 42.53–63.73%, *p* = 0.55); 36 weeks, AUC 59.58 (95% CI 49.66–69.69, *p* = 0.058); or 38 weeks, AUC 56.16 (95% CI 47.14–65.19%, *p* = 0.19) (Figure 4).

**Figure 5:**
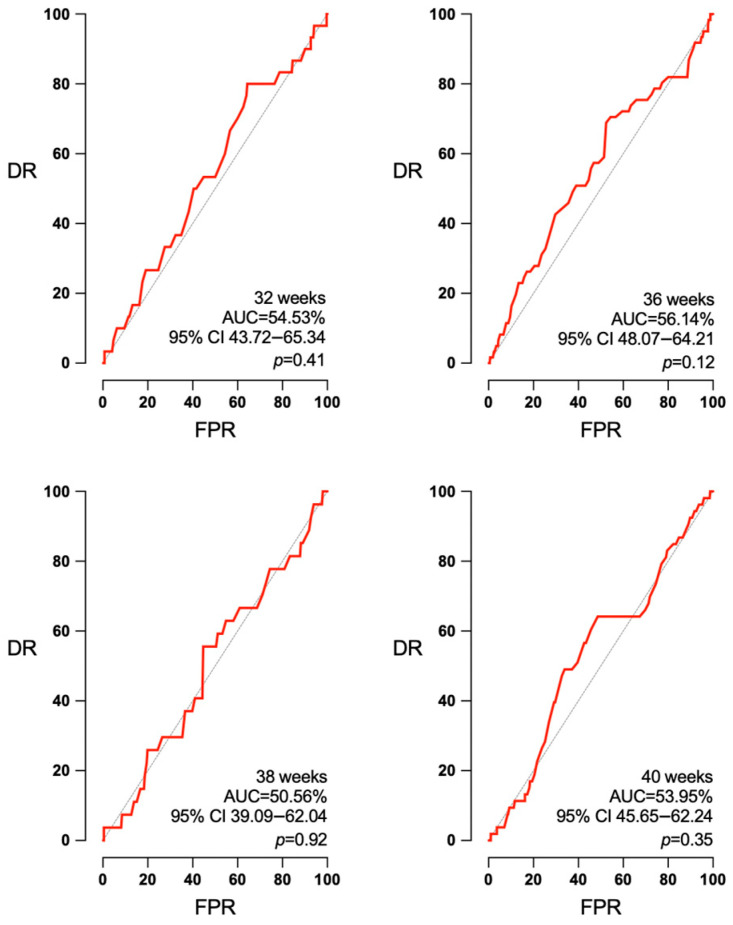
Absence of predictive capacity of UA PI independently of the week of pregnancy. AUC: area under the curve, CI: confidence interval, DR: detection rate, FPR: false positive rate, *p*: *p* value.

**Figure 6:**
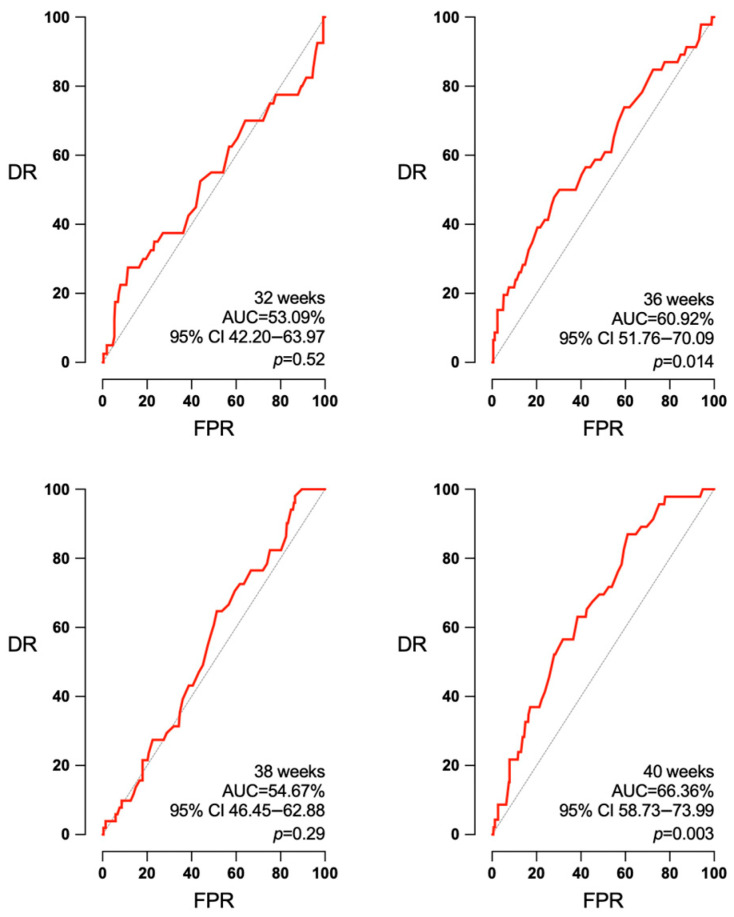
UA PI’s predictive capacity of SGA according to the interval to delivery. AUC: area under the curve, CI: confidence interval, DR: detection rate, FPR: false positive rate, *p*: *p* value.

Concerning the UA PI, it was not predictive of APO at any of the studied intervals (32, 36, 38 and 40 weeks): AUC 54.53 (95% CI 43.72–65.34%, *p* = 0.41), 56.14 (95% CI 48.07–64.21%, *p* = 0.12), 50.56 (95% CI 39.09–62.04%, *p* = 0.92), and 53.95 (95% CI 45.65–62.24%, *p* = 0.35) (Figure 5). UA PI could predict SGA at 36 weeks (AUC 60.92, 95% CI 51.76–70.09%, *p* = 0.014) and at 40 weeks (AUC 66.36, 95% CI 58.73–73.99%, *p* < 0.003). But it had no predictive ability at 32 weeks (AUC 53.09, 95% CI 42.20–63.97% *p* = 0.52) or at 38 weeks (AUC 54.67, 95% CI 46.45–62.88%, *p* = 0.29) (Figure 6).

## 4. Discussion

The results of this study show that the performance of one routine ultrasound in low-risk pregnancies at the late third trimester (36 weeks) has a similar detection rate for SGA to the performance of two scans in the mid- and late third trimester (32 and 38 weeks), with no increase in the risk of APO due to a diagnostic delay.

Low-risk pregnancies may be complicated by late-onset FGR, which is defined as that occurring beyond 32 weeks [19]. This condition is more common than early-onset FGR, and, although fetuses express only mild cerebral circulatory changes, consisting of an adaptative decrease of the cerebral impedance [20], they are reported to be at a higher risk of hypoxic distress [11], cerebral palsy [21], poorer neurodevelopment [2] and even neonatal death [22], especially in undiagnosed cases [23]. Furthermore, a large study has reported a significant reduction in fetal death when managed by a timely delivery around 37 weeks [24], showing the importance of the accurate detection and management of these babies.

Nevertheless, there continues to be a lot of debate regarding the impact of routine third-trimester ultrasonography on the reduction in APO. A meta-analysis by Bricker et al. [3] failed to demonstrate a benefit, although it has been argued [2] that, among other methodological problems, this might have been caused by the small proportion of trials that incorporated routine ultrasounds performed after 34 weeks of gestation (only 12% of the patients), when FGR is more likely to appear in low-risk pregnancies. Indeed, a large prospective study reported that universal screening triples the detection rate of SGA compared with screening based on clinical factors [7], and some studies showed that the performance of the routine third-trimester ultrasound improved in the late third trimester [9,10,25]. Accordingly, in our study, we observed that performing one ultrasound in the late third trimester (36 weeks) had a similar detection rate for SGA than performing two ultrasounds in mid and late third trimester (32 and 38 weeks), indicating the limited benefit of performing an ultrasound in low-risk pregnancies before the debut of late-onset FGR.

Interestingly, in our study, we observed that, when performed close to delivery (38 weeks), only CPR had a predictive ability to show APO. This is in accordance with a sizable body of evidence showing that UA Doppler does not reflect the placental insufficiency in late FGR, while CPR, which reflects the fetal hemodynamic adaptative changes to hypoxia, correlates better with APO [26,27,28,29,30]. Indeed, in a recent randomized controlled trial [13], the addition of CPR measurement to routine 36-week ultrasound reduced severe morbidity by 17%, showing the promising benefits of adding the information provided by Doppler to routine fetal growth assessment. Although this addition might not mean an increment in the costs of routine third-trimester ultrasound in high income countries, where most clinicians are trained to perform it, the costs of implementing this exam in underserved settings has not yet been studied.

The main strength of this study is the assessment in unison of two growth-scan-surveillance protocols, including all the extra ultrasounds performed for each pregnancy. This longitudinal perspective guaranteed a comparison between both protocols in terms of cost-effectiveness considering all the confusion variables. Furthermore, our study established evidence regarding the importance of adding Doppler ultrasound, and specifically CPR, to improve the prediction of APO when performing third trimester ultrasound in low-risk pregnancies. We acknowledge some limitations in our study. First, the retrospective nature of our cohort, despite the prospective collection of data, might have introduced some selection bias. Second, the fact that Doppler studies were only available in a proportion of cases might have underpowered to some extent the predictive ability reported. Therefore, a prospective study comparing low-risk pregnancy ultrasound at 36 weeks with and without CPR for the detection of APO shall be interesting to improve the evidence on this matter.

## 5. Conclusions

In conclusion, although our study shows the overall limitation of the routine third-trimester ultrasound for the prediction of SGA and APO, this seems to be improved by reducing the interval to labor and by the addition of Doppler. In addition, it demonstrates that the higher costs of a two-scan growth-surveillance protocol at the third trimester are not followed by an increase in diagnostic efficacy.

In accordance with our findings, routine third trimester ultrasound should follow a simplified approach that only included EFW and CPR at 36 weeks of pregnancy.

## Figures and Tables

**Table 1:** Study population descriptive analysis, n = 1011.

Parameter (Continuous Data)	Mean (SD)	Median (Q1–Q3)
Maternal age (years)	31.61 (5.20)	32 (28–36)
Gravidity	2.04 (1.18)	2 (1–3)
Parity	0.67 (0.82)	1 (0–1)
Maternal pre-pregnancy weight (Kg)	63.68 (11.5)	62 (55–70)
Maternal height (cm)	163 (6.18)	163 (159–167)
Maternal Body Mass Index	23.94 (3.95)	23.15 (21.08–25.89)
Gestational age at delivery (weeks)	39.76 (1.16)	39.86 (39.14–40.71)
Birth weight (g)	3309 (427.5)	3320 (3030–3600)
Birth weight centile	47.6 (29.8)	46 (21–73)
**Parameter (categorical data)**		n (%)
Nulliparity		498 (49.26)
Smoking		128 (12.66)
Male sex		516 (51.04)
Caucasian ethnicity		964 (95.35)
Type of labor onset		
Spontaneous onset of labor		579 (57.27)
Induction of labor		373 (36.89)
Elective cesarean section		59 (5.83)
Via of delivery		
Spontaneous vaginal delivery		615 (60.83)
Assisted vaginal delivery		198 (19.58)
Cesarean section (elective)		69 (6.83)
Cesarean section (failure to progress)		72 (7.12)
Cesarean section (abnormal CTG)		57 (5.64)
Apgar < 7 at 5 min		3 (0.3)
Arterial pH < 7.10		27 (2.67)
Neonate destiny		
Maternal ward		961 (94.06)
Neonates ward		49 (4.85)
Pediatric Intensive care unit		1 (0.1)
Adverse perinatal outcome		113 (11.17)
Abnormal CTG		57 (5.64)
Small for gestational age fetuses (<10th centile)		119 (11.77)

cm: centimeters, CTG: cardiotocogram, g: grams, Kg: kilograms, Q: quartile, n: numbers, SD: standard deviation.

**Table 2:** Descriptive analysis of the study population according to the ultrasonographic surveillance protocol followed.

	P2 32/38 Week Protocol (n = 528)	P1 36 Week Protocol (n = 483)	*p*-Value
	Mean (SD); Median (Q1–Q3)	Mean (SD); Median (Q1–Q3)	
Maternal age (years)	31.80 (5.10); 32 (29–35.75)	31.41 (5.3); 32 (28–36)	0.25
Gravidity	1.99 (1.17); 2 (1–2)	2.09 (1.19); 2 (1–3)	0.08
Parity	0.66 (0.79); 1 (0–1)	0.68 (0.86); 1 (0–1)	0.96
Maternal pre-pregnancy weight (Kg)	63.80 (11.21); 62 (56–70)	63.55 (11.88); 61 (55–70)	0.47
Maternal height (cm)	163.1 (6.28); 163 (159–181)	162.9 (6.07); 163 (159–167)	0.49
Maternal Body Mass Index	23.96 (3.92); 23.07 (21.22–26.16)	23.9 (3.98); 23.34 (20.9–25.8)	0.75
Gestational age at delivery (weeks)	39.73 (1.23); 39.86 (39.14–40.71)	39.8 (1.07); 40 (39.14–40.71)	0.65
Birth weight (g)	3284 (448.4); 3300 (3000–3580)	3336 (402.1); 3350 (3075–3640)	0.06
Birth weight centile	46.15 (30.39); 45 (17–72)	49.18 (29.09); 47 (24–75)	0.09
	**n (%)**	**n (%)**	
Nulliparity	257 (48.67)	241 (49.9)	0.71
Smoking	72 (13.64)	56 (11.59)	0.34
Male sex	258 (48.86)	258 (53.42)	0.16
Caucasian ethnicity	514 (97.35)	450 (93.17)	<0.0024
Type of labour onset			
Spontaneous onset of labor	319 (60.42)	260 (53.83)	0.03
Induction of labor	173 (32.77)	200 (41.41)	<0.005
Elective cesarean section	36 (6.82)	23 (4.76)	0.18
Via of delivery			
Spontaneous vaginal delivery	316 (59.85)	299 (61.9)	0.52
Assisted vaginal delivery	106 (20.08)	92 (19.05)	0.69
Cesarean section (elective)	41 (7.77)	28 (5.8)	0.26
Cesarean section (failure to progress)	39 (7.39)	33 (6.83)	0.81
Cesarean section (abnormal CTG)	26 (4.92)	31 (6.42)	0.34
Apgar < 7 at 5 min	1 (0.19)	2 (0.41)	0.61
Arterial pH < 7.10	8 (1.52)	19 (3.93)	<0.019
Neonate destiny			
Maternal ward	500 (94.70)	461 (95.44)	0.66
Neonates ward	27 (5.11)	22 (4.55)	0.80
Pediatric Intensive care unit	1 (0.19)	0	>0.99
Adverse perinatal outcome	51 (9.66)	62 (12.84)	0.11
Abnormal CTG	26 (4.92)	31 (6.42)	0.34
Small for gestational age fetuses (<10th centile)	71 (13.45)	48 (9.94)	0.10

cm: centimeters, CTG: cardiotocogram, g: grams, Kg: kilograms, Q: quartile, n: numbers, SD: standard deviation.

## Data Availability

The data from this study are available upon request.
